# Temperature effects from hydropeaking impair egg and larval development in Danube salmon *Hucho hucho* and brown trout *Salmo trutta*

**DOI:** 10.1111/jfb.70501

**Published:** 2026-05-17

**Authors:** Julius Gorenz, Elisabeth Winter, Joachim Pander, Juergen Geist

**Affiliations:** ^1^ Aquatic Systems Biology Unit, School of Life Sciences Technical University of Munich Freising Germany

**Keywords:** environmental flow, fish egg bioindication, fish stock management, hydropower, spawning ground

## Abstract

Hydropeaking is considered an important stressor for salmonids and other species in rivers affected by hydropower. In this study, we investigated the *in situ* effects of hydropeaking on the survival of eggs and larvae of fall spawning brown trout as well as spring spawning Danube salmon. Egg‐to‐fry survival of these two species decreased with increasing time of dewatering and desiccation, resulting in temperature effects of overheating and freezing of eggs and larvae. Consequently, mitigation measures should aim for slow ramping rates and sufficient water cover of spawning grounds.

Salmonids are subject to multiple stressors in anthropogenically altered aquatic ecosystems, including but not limited to the loss, degradation and separation of key habitats. This impact is further intensified by warming waters and shifting flow regimes caused by global climate change with a projected reduction in available habitat space (Aarts et al., 2004; Smialek et al., 2021; Wild et al., 2023, 2024).

At the same time, rising energy demand and a shift towards renewable sources foster the expansion of demand‐responsive hydropower generation (Geist, 2021; Zarfl et al., 2015), resulting in rapid periodic dewatering and flooding of downstream reaches, referred to as hydropeaking. These unnatural changes in discharge can result in detrimental ecological impacts on riverine species, including reduced habitat availability (e.g., Boavida et al., 2015; Freeman et al., 2001), stranding and displacement of larvae and juvenile fish (e.g., Auer et al., 2023; Bauersfeld, 1976; Führer et al., 2024; Hayes et al., 2025; Nagrodski et al., 2012) and invertebrates (e.g., Bruno et al., 2010; Perry & Perry, 1986) as well as increased mortality of fish eggs and larvae of fall spawning species as a consequence to low oxygen supply (e.g., Pander et al., 2023) or desiccation and exposure to freezing temperatures (Casas‐Mulet et al., 2015). While impacts of hydropeaking on later developmental stages of fish are well studied, effects on interstitial phases of eggs and yolk sac larvae are so far underrepresented and not systematically investigated in real‐world scenarios across multiple species, spawning seasons and associated environmental temperatures.

To fill this gap, we investigated the *in situ* effects of hydropeaking‐related dewatering on the survival of eggs and larvae of fall spawning brown trout (*Salmo trutta* L. 1758) as well as spring spawning Danube salmon (*Hucho hucho* [L.]). Our study took place at the River Ziller (spring trial: mean discharge 55 ± 22 m^3^s^−1^; winter trial: 28 ± 21 m^3^s^−1^), approximately 500 m from its confluence with the River Inn. Six hydropower plants are located in the alpine catchment of the River Ziller with a combined capacity of 965 MW, leading to a pronounced hydropeaking regime in the downstream reach, with over 63 m^3^s^−1^ maximum hourly change in discharge during our study (Figure 1). In an active bioindication experiment, we used 720 eye‐staged brown trout *Salmo trutta* eggs in 20 modified egg‐sandwiches (ES) (Pander et al., 2009), and due to their shorter developmental time and high conservation status, 180 freshly fertilised eggs of Danube salmon *Hucho hucho* in 5 ES. The Danube salmon ES were exposed for 29 days (19th of April to the 17th of May 2024) and the brown trout ES for 78 days (27th of November 2024 to the 12th of February 2025) in the substrate of a predetermined spawning ground, represented by a large, submerged gravel bank located within a steep riffle section. The main substrate was gravel with 34% grain sizes <20 mm and 3.9% of fines (diameter <0.85 mm; Table S1) in spring and 26% <20 mm and 1.8% of fines in fall. The general suitability of the selected spawning ground was determined preceding ES deposition, as done in Gorenz et al. (2026) by measurements of physicochemical and hydraulic conditions of the free drain and the interstitial water. In particular, measurements of water depth and current speed as well as substrate samples (Pander et al., 2015; Table S1) were used for spawning ground selection by comparing them to values reported for functional salmonid spawning grounds in the literature (reviewed in Smialek et al., 2021). In addition, this site was known for historical records of spawning activity of Danube salmon and brown trout. The location of the ES represented natural redd positions across the depth profile of brown trout and Danube salmon spawning grounds according to our physicochemical measurements. To mimic natural egg deposition, ES (dimensions: 12 × 7.5 × 1.5 cm length × width × height) were fixed upright in the riverbed using metal rods, covered with a thin layer of gravel (3–5 cm) and spaced along the ramping of the spawning ground, resulting in egg burial depths ranging from approximately 3–17 cm. Water depths of the free drain above the buried ES at deposition were between 18 cm and 45 cm, covering a lateral distance of 5 m (Danube salmon) to 7.5 m (brown trout) across the part of the river bed potentially functional for spawning (Table S1). The discharge of the River Ziller at ES deposition with Danube salmon was around 50 m^3^s^−1^ at a daily mean of 62 ± 20 m^3^s^−1^ and 20 m^3^s^−1^ with brown trout at a daily mean of 32 ± 19 m^3^s^−1^. Due to their higher quantity, ES loaded with brown trout eggs were compiled into 4 groups along this depth gradient. At the end of our trial, ES were extracted from the riverbed and analysed, as done in Pander et al. (2009). As a reference, 300 Danube salmon eggs and 900 brown trout eggs were kept under stable pumped ground water conditions in up‐flow incubation trays at the hatchery of the Aquatic Systems Biology Unit of TUM (Freising, Germany). All experiments were conducted following the German animal protection and welfare legislation. Since only stages of eggs and larvae before consumption of the yolk sack were used, no animal experiment application was required according to the national ‘§14 Tierschutzversuchstierverordnung’ and all experiments passed a voluntary inspection of the Aquatic Systems Biology Animal Welfare Committee at Technical University of Munich.

**FIGURE 1:**
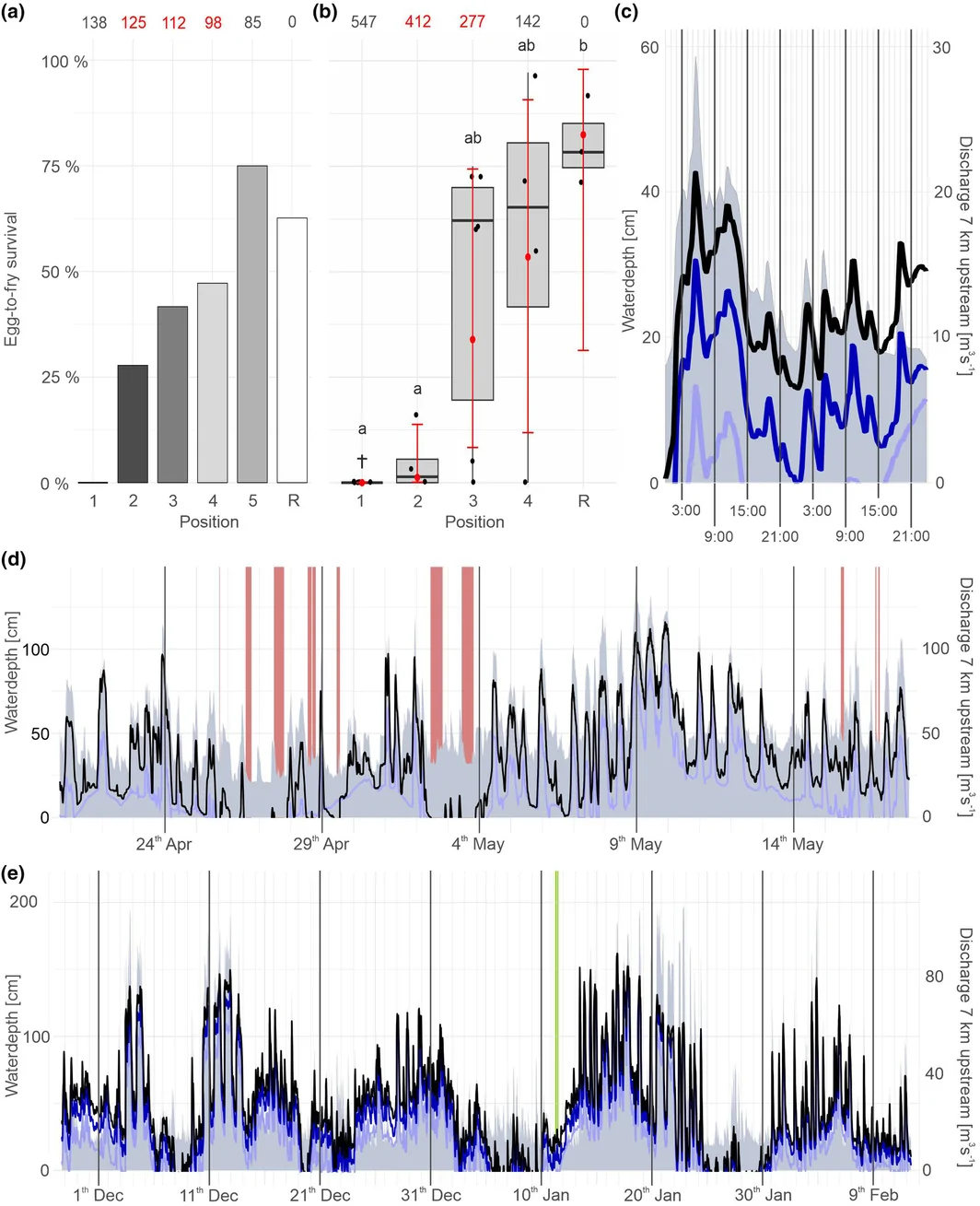
Egg‐to‐fry survival of Danube salmon *Hucho hucho* (a) and brown trout *Salmo trutta* (b) along the ramping of a spawning ground from shore to river centre (position 1–4/5) in a hydropeaking‐affected river and a hatchery reference (R). Boxplots summarise the distribution of observed egg‐to‐fry survival proportions within each egg‐sandwich (ES) bioindication tool, with individual ES shown as black jitter points. Red dots denote predicted survival probabilities estimated from a binomial GLMM, with 95% confidence intervals shown as red error bars. †Boundary case: For groups with complete separation (0% survival), the asymptotic upper confidence interval exceeded the biologically meaningful probability range. For clarity, upper CIs were omitted in these cases. Different lower‐case letters indicate significant differences (*p* < 0.05). Numbers above bars/boxes indicate the hours each position was exposed to dry conditions. Black values: Measured data, red values: Data derived from linear interpolation. Panel (c) depicts the measured water depths across the spawning ground (black, dark blue and purple lines) and the discharge of the Ziller River (grey area) of a typical period of down ramping resulting in desiccation of parts of the spawning ground (here: 10th and 11th of January 2025). Water depth across the spawning ground and discharge of the River Ziller during the whole interstitial exposition of Danube salmon eggs in spring 2024 and brown trout in winter 2024/2025 are displayed in panels (d) and (e). Black, dark blue and purple lines depict water depth values closest to the shore, in the middle and closest to the river centre of the spawning ground. In spring 2024 (d), only the outer loggers were placed. The grey area depicts the discharge measured at a water gauge station 7 km upstream (data supplied by Land Tyrol). Red and green bars indicate time intervals of combined desiccation of the spawning ground and temperatures >18°C (red) or desiccation and temperatures ≤0°C.

To monitor water levels and temperatures during the development of eggs and larvae in our trial, we introduced HOBO Onset U20L‐04 loggers together with the ES into the spawning ground, measuring water pressure at the lower end of the ES. Due to the smaller scale of the Danube salmon trial in spring, we used 2 loggers, one at the bank and one at the river site of the spawning ground, set to 10‐min logging intervals. In fall and winter (brown trout trial), we added an additional logger to the middle of the spawning ground and measured at 15‐min intervals.

Water levels were calculated from the recorded pressure values following the formula:

$$d=\left(p+\left(∆p_{\mathrm{atm}}-p_{\mathrm{atm}\_\text{inst}}\right)\right)\times 0.0980665$$

$$d=\text{depth}\;\left[\mathrm{cm}\right]$$

$$p=\text{pressure}\;\left[\mathrm{kPa}\right],\text{recorded}\;\mathrm{by}\;\text{the logger}$$

$$p_{\mathrm{atm}\_\text{inst}}=\text{Atmospheric pressure}\;\left[\mathrm{kPa}\right]\;\mathrm{at}\;\text{installation}$$

$$∆p_{\mathrm{atm}}=\text{Changes in atmospheric pressure over the trial}$$

Changes in atmospheric pressure throughout the trials were calculated from data supplied by the closest weather station in Jenbach, Austria 6 km away from our sampling site (www.geosphere.at). Since the HOBO loggers need to perform an internal temperature calibration for their pressure readings and logger temperatures change fast if loggers are out of the water and exposed to direct sunlight, depth readings below zero did not represent actual pressure measurements and were therefore excluded from the visualisation of the depth data.

For the analysis of the variation in egg‐to‐fry survival of brown trout along the ramping of the spawning ground and comparison to the values attained by the hatchery reference, we fitted a generalised linear mixed model (GLMM) with binomial error distribution and a logit link. Survival was expressed as the number of eggs and larvae alive versus dead per ES, modelled using the formula *cbind(sum_alive, sum_dead) ~ ID_position_group + (1|ID_Flake)*. Model diagnostics were performed using the DHARMa package. Residual simulations showed no evidence of overdispersion, no zero‐inflation and no influential observations, indicating that the GLMM adequately captured the variance structure and distributional properties of the data. Post‐hoc pairwise comparisons were performed on estimated marginal means (emmeans package in R) using Tukey‐adjusted contrasts. Due to the limited sample size, the analysis of variation in survival in the Danube salmon trial was constrained to the utilisation of descriptive statistics.

Out of 29 days, Danube salmon eggs and larvae were without water cover between 85.6 and 138.3 h (13.2%–21.7% of logged depths), depending on their position across the spawning ground, with five times dry periods lasting over 6 h and a maximum duration in a desiccated state of 21.1 to 45.8 h. Desiccation did not occur in combination with freezing temperatures for spring spawning Danube salmon, but did coincide with temperatures above 18°C on 7 days, with a maximum spawning ground temperature of 40.9°C. (Figure 1). During the 78 days of brown trout eggs and larvae exposure in the interstitial, ES ran dry between 142.5 and 547.0 h (8.1%–30.5% of logged depths), with dry periods lasting over 6 h 9 to 21 times and with a maximum duration of 41.0 to 78.5 h. Combined exposure to dry conditions and temperatures below 0°C were recorded on 19 different days with a minimum temperature of −0.3°C (Figure 1). The minimum discharge during the spring trial was at 20.2 m^3^s^−1^, 34.6 m^3^s^−1^ below the mean discharge and during the winter trial at 8.7 m^3^s^−1^, 19.8 m^3^s^−1^ below the mean. When not dried out, interstitial water parameters of the spawning ground remained stable throughout the trials with DO values above 9 mgL^−1^.

Mortality of eggs and larvae of both species followed the ramping of the spawning ground and the resulting increase in frequency and duration of desiccated phases from complete mortality in the shallowest part of the spawning ground to 75.0% survival for Danube salmon and 96.7% for brown trout (hatchery reference survival: Danube salmon 62.6%; brown trout 80.4%) (Figure 1). A binomial GLMM showed that the survival of brown trout differed significantly among the groups of ES spaced along this gradient and the hatchery reference (*χ* ^2^‐test vs. null model, *p* < 0.001), with a strong overall effect of the position on the survival of eggs and larvae. Estimated marginal means indicated low survival probabilities in the two groups closer to the bank (0.0%–1.2%) and over 10‐fold higher survival probabilities in groups 3 and 4 further in the river (33.9%–53.5%), compared to an estimated survival probability in the hatchery reference of 82.5%. Tukey‐adjusted pairwise tests showed that only Groups 1 and 2 differed significantly from the reference (both *p* < 0.001); all other pairwise comparisons were non‐significant.

A combination of dry conditions and freezing temperatures has been identified as the primary cause of mortality among Atlantic salmon (*Salmo salar*) eggs in hydropeaking conditions in Norway (Casas‐Mulet et al., 2015). This stressor combination was observed during the interstitial development of eggs and larvae of fall spawning brown trout, but not for spring spawning Danube salmon. Nevertheless, we observed a similar mortality gradient along the ramping of the spawning ground in spring. During the spring trial, the desiccation of eggs occurred concurrently with spawning ground temperatures lethal for salmonid eggs (>18°C; reviewed in Smialek et al., 2021) on multiple days and with a temperature extreme of 40.9°C, strongly suggesting that the observed mortality in the spring spawning species was due to the prolonged periods of low flow in combination with elevated radiation and temperature levels in spring. Although longer developmental times and naturally lower discharge during winter in meltwater fed alpine rivers might lead to a higher potential risk of desiccation and mortality of eggs and larvae of fall spawning species, our findings demonstrate that early developmental phases of spring spawners are similarly threatened by hydropeaking‐related dewatering. Particularly, since gill‐breathing larval phases are more sensitive to low water supply compared to incubated eggs (Becker et al., 1982; Casas‐Mulet et al., 2016), and 65% of living Danube salmon did not hatch until the end of the experiment. Mortalities might therefore even be higher when considering the whole interstitial developmental phase.

The Danube salmon is a species of high conservation interest nowadays, with limited natural reproduction in the River Inn network. Reported observations of Danube salmon spawning activities in the River Ziller by local anglers underline the importance of immediate mitigation measures in this important tributary in the upper catchment of the River Inn. These measures need to mitigate fast down‐ramping rates to minimise stranding of eggs, larvae and juveniles as well as long periods of insufficient water cover of redds, to avoid freezing or overheating of eggs and larvae for fall and spring spawners alike. From a conservation perspective, artificial down ramping should be suspended entirely during the spawning and incubation season of Danube salmon if critical water levels are still not known for all spawning sites in the system.

## AUTHOR CONTRIBUTIONS

*Conceptualisation*: Julius Gorenz, Elisabeth Winter and Joachim Pander and Juergen Geist. *Data Curation*: Julius Gorenz and Elisabeth Winter. *Funding Acquisition*: Joachim Pander and Juergen Geist. *Investigation*: Julius Gorenz, Elisabeth Winter, Joachim Pander and Juergen Geist. *Methodology*: Julius Gorenz, Elisabeth Winter, Joachim Pander and Juergen Geist. *Project Administration*: Juergen Geist. *Resources*: Juergen Geist. *Supervision*: Juergen Geist. *Validation*: Joachim Pander and Juergen Geist. *Visualisation*: Julius Gorenz. *Writing—Original Draft Preparation*: Julius Gorenz. *Writing—Review & Editing*: Elisabeth Winter, Juergen Geist and Joachim Pander.

## FUNDING INFORMATION

This study was partly funded by the ‘EU‐Interreg VI‐A Bayern‐Österreich’ via the Project ‘INNsieme connect BA0100108’.

## Supporting information


**Table S1.** Ecologically relevant parameters of the hyporheic zone, fine sediment content (FC) of the substrate and water depths at egg‐sandwich deposition of the spawning ground in the River Ziller across spring and winter trials with Danube salmon *Hucho hucho* and brown trout *Salmo trutta*, respectively, and flow velocities and water depths at egg‐sandwich deposition, as well as hatchery reference values.

## Data Availability

The data that support the findings of this study are available from the corresponding author upon reasonable request.
