# Supplementary material for: Temperature effects from hydropeaking impair egg and larval development in Danube salmon Hucho hucho and brown trout Salmo trutta
Source: J Fish Biol. 2026 May 17;109(2):1312–6. doi: 10.1111/jfb.70501 (PMC13521711; doi:10.1111/jfb.70501)
Supplement: Supplementary file 1 [file JFB-109-1312-s001.docx]

**Supporting Information**

***Journal of Fish Biology***

**Temperature effects from hydropeaking impair egg and larval development in Danube salmon *Hucho hucho* and brown trout *Salmo trutta***

Julius Gorenz, Elisabeth Winter, Joachim Pander, Juergen Geist

Aquatic Systems Biology Unit, School of Life Sciences,

Technical University of Munich, Mühlenweg 22, D-85354 Freising, Germany

**Corresponding author**

Juergen Geist

Aquatic Systems Biology, School of Life Sciences, Technical University of Munich, Mühlenweg 22, D-85354 Freising, Germany

Email: geist@tum.de

Phone: +49-8161-713767

Fax: +49-8161-713477

**Funding**

This study was partly funded by the “EU-INTERREG VI-A Bayern – Österreich” via the Project “INNsieme connect BA0100108”.

| **Season** | **Species** | **Site** | **Parameter** | **Mean ± SD** | **Median** | **Range** |
| --- | --- | --- | --- | --- | --- | --- |
| Spring | **Danube salmon** | Spawning ground | T (°C) | 12.02 ± 2.14 | 12.20 | 8.50 – 14.40 |
|  |  |  | DO (mg.L^-1^) | 9.79 ± 0.52 | 9.78 | 9.12 – 10.51 |
|  |  |  | pH | 8.97 ± 0.15 | 9.00 | 8.63 – 9.13 |
|  |  |  | EC (μS.cm^-1^) | 116.70 ± 14.51 | 116.70 | 99.20 – 140.80 |
|  |  |  | FC (%_weight_) | 3.91 ± 2.42 | 3.17 | 1.37 – 8.93 |
|  |  |  | Flow velocity at ES deposition (m.s^-1^) | 0.88 ± 0.26 | 0.83 | 0.52 – 1.45 |
|  |  |  | Water depth at ES deposition (m) | 0.31 ± 0.1 | 0.30 | 0.18 – 0.45 |
|  |  | Hatchery | T (°C) | 12.33 ± 0.06 | 12.30 | 12.30 – 12.40 |
|  |  |  | DO (mg.L^-1^) | 9.96 ± 0.01 | 9.96 | 9.95 – 9.97 |
|  |  |  | pH | 8.97 ± 0.15 | 9.00 | 8.63 – 9.13 |
|  |  |  | EC (μS.cm^-1^) | 1281.67 ± 4.73 | 1280.00 | 1278.00 – 1287.00 |
| Winter | **Brown trout** | Spawning ground | T (°C) | 4.43 ± 0.65 | 4.65 | 3.00 – 5.00 |
|  |  |  | DO (mg.L^-1^) | 12.31 ± 0.56 | 12.18 | 11.71 – 13.33 |
|  |  |  | pH | 8.27 ± 0.22 | 8.26 | 8.01 – 8.82 |
|  |  |  | EC (μS.cm^-1^) | 139.63 ± 36.53 | 138.30 | 95.90 – 187.20 |
|  |  |  | FC (%_weight_) | 1.80 ± 0.99 | 1.57 | 0.46 – 4.21 |
|  |  |  | Flow velocity at ES deposition (m.s^-1^) | 0.71 ± 0.15 | 0.71 | 0.50 – 0.91 |
|  |  |  | Water depth at ES deposition | 0.35 ± 0.05 | 0.36 | 0.30 – 0.40 |
|  |  | Hatchery | T (°C) | 11.78 ± 0.16 | 11.85 | 11.50 – 11.90 |
|  |  |  | DO (mg.L^-1^) | 9.86 ± 0.38 | 9.97 | 9.22 – 10.29 |
|  |  |  | pH | 8.97 ± 0.15 | 9.00 | 8.63 – 8.16 |
|  |  |  | EC (μS.cm^-1^) | 1180.67 ± 12.88 | 1179.00 | 1164.00 – 1197.00 |

Table S1 Ecologically relevant parameters of the hyporheic zone, fine sediment content (FC) of the substrate and water depths at egg-sandwich deposition of the spawning ground in the River Ziller across spring and winter trials with Danube salmon Hucho hucho and brown trout Salmo trutta respectively and flow velocities and water depths (from water surface to river bottom) at egg-sandwich deposition, as well as hatchery reference values. T = temperature; DO = dissolved oxygen concentration; EC = electric conductivity; SC = sediment compaction; FC = fine sediment content.
